# Efficacy of artificial intelligence-based FFR technology for coronary CTA stenosis detection in clinical management of coronary artery disease: a systematic review

**DOI:** 10.3389/fphys.2025.1635923

**Published:** 2025-07-31

**Authors:** Tong Liu, Ming Liu, Ailiyaerjiang Aisika, Palidanmu Wumaier, Abudukeyoumujiang Abulizi, Jingru Wang, Mayidili Nijiati

**Affiliations:** ^1^ Department of Radiology, Medical Imaging Center, Xinjiang Medical University Affiliated Fourth Hospital, Urumqi, China; ^2^ Department of Radiology, The First People’s Hospital of Kashi Prefecture, Kashi, China; ^3^ Xinjiang Key Laboratory of Artificial Intelligence assisted Imaging Diagnosis, Kashi, China; ^4^ Department of Deepwise AI Lab, Hangzhou Deepwise & League of PHD Technology Co., Ltd, Hangzhou, China

**Keywords:** artificial intelligence, fractional flow reserve (FFR), coronary computed tomography angiography (CCTA), coronary artery disease, stenosis detection, deep learning, AI-assisted quantitative coronary CTA (AI-QCT) based coronary artery bypass grafting

## Abstract

Coronary computed tomography angiography (CCTA) integrated with artificial intelligence (AI) technology, particularly AI-based fractional flow reserve (FFR) assessment, has emerged as a crucial tool in the diagnosis and treatment of coronary artery disease (CAD). Recent advances in AI technology have demonstrated promising applications of AI-based FFR in detecting coronary stenosis through CCTA. Current evidence suggests that AI-FFR offers significant advantages in diagnostic accuracy and clinical utility, potentially enhancing the efficiency of CAD management. However, challenges persist in algorithm robustness, data heterogeneity, and clinical implementation. This review synthesizes recent developments in AI-based FFR technology for coronary stenosis detection via CCTA, focusing on AI-assisted quantitative coronary CTA (AI-QCT), deep learning algorithms, and their applications in three-dimensional coronary reconstruction and hemodynamic simulation. We analyze comparative diagnostic performance between AI-FFR and conventional methods, providing insights for future research directions and clinical applications.

## 1 Introduction

Coronary artery disease (CAD) represents one of the leading causes of mortality worldwide, with continuously increasing incidence and mortality rates. Early and accurate diagnosis of coronary stenosis severity is crucial for establishing appropriate clinical treatment strategies. Traditional diagnostic methods, including invasive fractional flow reserve (FFR) and quantitative coronary angiography (QCA), are considered the gold standards for evaluating coronary stenosis. However, these approaches are associated with procedural risks and substantial costs, limiting their widespread application in routine clinical practice.

In recent years, coronary computed tomography angiography (CCTA) has been increasingly utilized in CAD diagnosis due to its non-invasive nature and high spatial resolution. However, quantitative assessment of stenosis severity using CCTA often relies on the interpreting physician’s experience, introducing a degree of subjective error that may compromise the accuracy of clinical decision-making (Guo et al., 2024).

The introduction of artificial intelligence (AI) has provided novel solutions to address these challenges. Deep learning-based FFR computation methods (CT-FFR) can enhance the accuracy of stenosis assessment by extracting critical features from CCTA images while minimizing human interference (Li B. et al., 2024; Wu et al., 2025). Recent studies have demonstrated that AI-driven automated CT-FFR technology significantly improves computation success rates and reduces processing time in clinical applications (Guo et al., 2024). This advancement has enabled automated analysis of complex vascular geometries and hemodynamic parameters, providing comprehensive quantitative assessments that closely correlate with invasive FFR measurements.

For instance, state-of-the-art research has shown that AI-based systems can automatically analyze complex vascular geometries and hemodynamic parameters, providing comprehensive quantitative assessments that closely correlate with invasive FFR measurements. These automated systems not only enhance diagnostic accuracy but also offer the potential for standardized evaluation protocols across different clinical settings, thereby reducing inter-observer variability and improving the consistency of diagnostic outcomes.

Furthermore, the integration of AI technology into coronary CTA has facilitated the identification of high-risk plaque characteristics, including thin fibrous caps, lipid-rich necrotic cores, and microcalcifications, which are strongly associated with acute coronary events (Wu et al., 2025). By combining CT-FFR with plaque characterization, clinicians can conduct more comprehensive risk assessments and develop personalized therapeutic strategies for patients with coronary artery disease.

### 1.1 Current status of AI-Based quantitative analysis of coronary CTA

In recent years, artificial intelligence (AI)-based coronary computed tomography angiography (CTA) has transformed the diagnosis and management of coronary artery disease. The integration of AI technologies has markedly enhanced both the efficiency and accuracy of quantitative coronary CTA analysis, particularly in assessing coronary stenosis severity and predicting myocardial ischemia. While conventional coronary CTA interpretation typically relies on experienced radiologists, making it time-consuming and subject to interobserver variability, AI-based solutions enable rapid, objective data analysis, substantially improving diagnostic accuracy and consistency.

A recent investigation introduced a novel, fully automated on-site CT-FFR technology that demonstrated significant advances in clinical implementation. This technology combines automated coronary plaque segmentation and lumen extraction models with a simplified three-dimensional computational fluid dynamics model. The study evaluated 600 vessels in 463 patients and achieved an impressive accuracy of 0.82. Notably, the computational process was streamlined, reducing operational steps from approximately 60 to just one, making it substantially more practical for clinical use (Guo et al., 2024).

Further validation of AI technologies’ robust diagnostic performance came from a study assessing 67 target vessels in 40 patients with stable coronary artery disease. By integrating CT-FFR with AI-assisted quantitative coronary CTA analysis, the research demonstrated that AI-QCT achieved a diagnostic accuracy of 92.54%, with exceptional sensitivity and specificity for identifying ischemic lesions (Xue et al., 2022). This approach proves particularly valuable as it provides accurate diagnostic information promptly without increasing patient burden.

The technical implementation of AI extends beyond basic image interpretation to include comprehensive quantitative assessment of coronary plaques. AI algorithms can now identify and quantify various plaque characteristics, including volume and density, providing crucial data for clinical decision-making. Recent studies have shown that AI analysis based on CT-FFR demonstrates significant prognostic value, particularly in evaluating cardiovascular event risk, where AI-QCT models exhibit excellent accuracy and reliability (Nurmohamed et al., 2024). These advancements represent a significant step forward in the objective and efficient diagnosis of coronary artery disease.

#### 1.1.1 AI-QCT: Principles and workflow

Artificial Intelligence-based Quantitative Computed Tomography (AI-QCT) represents a cutting-edge technology that integrates deep learning algorithms with coronary imaging to achieve automated coronary artery segmentation, lumen and wall thickness measurements, and quantitative plaque analysis. Through efficient algorithms, this technology extracts critical information from complex CT images, thereby supporting the diagnosis and management of coronary artery disease.

Initially, AI-QCT technology employs deep learning models for automated coronary artery segmentation. This process primarily relies on convolutional neural networks (CNNs), which, unlike traditional image processing methods, can autonomously learn and extract features from images, thereby achieving precise vessel identification. Research demonstrates that deep learning-based automated segmentation methods significantly enhance accuracy and efficiency when processing complex coronary images (Guo et al., 2024; Carvalho et al., 2024).

Subsequently, AI-QCT technology facilitates measurements of vessel lumen and wall thickness. Through three-dimensional reconstruction of coronary arteries, the system precisely calculates the diameter and wall thickness of individual vessels, establishing the foundation for further plaque analysis. Relevant studies indicate that AI-QCT technology provides superior resolution and accuracy in lumen and wall thickness measurements compared to conventional methods, facilitating early detection of potential coronary lesions (Guo et al., 2024; Xue et al., 2022).

Furthermore, quantitative plaque analysis constitutes a critical function of AI-QCT. This technology evaluates plaque volume, composition, and its impact on the vessel lumen. Automated plaque analysis not only enhances analytical efficiency but also minimizes human error, ensuring result consistency and reliability. Research indicates that plaque analysis via AI-QCT achieves greater accuracy than traditional manual analytical methods, providing clinicians with more objective data support (Guo et al., 2024; Xue et al., 2022).

Finally, the cloud-based platform of AI-QCT technology supports rapid automated analysis, with an average processing time of approximately 10 min. This improvement in speed significantly enhances clinical workflow efficiency, enabling physicians to obtain accurate imaging data within a short timeframe, thereby facilitating better individualized treatment planning for patients (Guo et al., 2024; Carvalho et al., 2024). This efficient analytical workflow demonstrates the immense potential of AI-QCT technology in early diagnosis of coronary artery disease and the formulation of treatment strategies.

#### 1.1.2 Primary algorithmic framework and technical innovations

Artificial intelligence-based coronary computed tomography angiography (CTA) technology demonstrates significant potential in the diagnosis and management of coronary artery disease. Recently, the integration of deep learning with level set algorithms has provided novel approaches for optimizing three-dimensional coronary reconstruction, thereby enhancing the precision of hemodynamic simulations. These technological innovations enable more effective analysis of complex vascular morphology and lesion characteristics in identifying coronary stenosis severity. For instance, deep learning models can automatically recognize varying degrees of stenosis and lesion types through processing large volumes of annotated data, significantly improving diagnostic accuracy and efficiency (Griffin et al., 2023; Koo et al., 2024).

Specifically, AI-QCT (Artificial Intelligence-based Quantitative Coronary Computed Tomography Angiography) employs advanced image processing algorithms, such as convolutional neural networks (CNNs), to conduct deep learning analysis of coronary CTA images. These models, through learning from extensive clinical data, can not only accurately identify stenosis severity but also classify lesion components, including plaque characteristics such as calcification degree and plaque stability (Lipkin et al., 2022; Jonas et al., 2025). These AI-based models demonstrate comparable or superior accuracy to traditional coronary CTA interpretation methods, particularly in identifying >50% or >70% stenosis, where AI-QCT exhibits high sensitivity (94%) and specificity (68%–82%) compared to conventional CCTA approaches (Griffin et al., 2023; Kero et al., 2025).

Furthermore, level set algorithms play a crucial role in three-dimensional coronary reconstruction. This methodology precisely delineates vascular boundaries through dynamic evolution, thereby enhancing the characterization of vascular morphology. Compared to traditional image processing techniques, level set algorithms demonstrate superior capability in processing complex vessel geometries, particularly in cases of stenosis or occlusion (Li B. et al., 2024; Jonas et al., 2025). The integration of level set algorithms with deep learning not only improves reconstruction precision but also provides a more reliable foundation for subsequent hemodynamic analysis.

AI has transformed coronary artery hemodynamic analysis, particularly in FFR assessment (Omori et al., 2023). Deep learning models can now quickly predict FFR-CT from coronary CTA images, matching the accuracy of invasive measurements (Giannopoulos et al., 2023). Machine learning algorithms analyze flow patterns and wall shear stress to identify atherosclerosis-prone regions (Li et al., 2016; Pattarabanjird et al., 2020). This integration of anatomical and functional assessments aids in treatment planning and risk stratification.

Overall, the continuous advancement of artificial intelligence-based FFR technology has enabled non-invasive coronary physiological measurements, thereby providing patients with safer and more efficient diagnostic modalities. These technological innovations offer new perspectives for the clinical management of coronary artery disease, and the future integration of AI with traditional imaging techniques will further advance precision diagnosis and treatment of cardiovascular diseases (Nakanishi et al., 2023; Andre et al., 2023).

#### 1.1.3 Current AI platforms and software applications

In the contemporary field of medical imaging, artificial intelligence (AI) technology applications are continuously expanding, particularly in CCTA and FFR assessment. Recently, FDA-approved AI-QCT software has demonstrated exceptional performance in multicenter clinical trials, indicating its potential for clinical implementation. This software leverages deep learning and image processing technologies to achieve automated coronary artery segmentation and plaque extraction, significantly enhancing diagnostic accuracy and efficiency.

In one study, AI-QCT technology was evaluated in 600 vessels from 463 patients, achieving a diagnostic accuracy of 0.82, with substantially reduced operation time compared to traditional semi-automated CT-FFR techniques, decreasing from approximately 60 clicks to a single click (Guo et al., 2024). This automated assessment approach not only saves considerable clinical time but also dramatically improves computational efficiency, with a calculation success rate exceeding 99%. Furthermore, AI-QCT demonstrated superior prognostic value in predicting major adverse cardiac events compared to CCTA alone, indicating its clinical efficacy in assessing coronary stenosis functionality.

Similarly, another study evaluated AI-assisted coronary functional assessment in patients with stable coronary artery disease by combining CCTA and invasive coronary angiography (FFR) methodologies. In a cohort of 40 patients with stable coronary artery disease, the correlation between AI-assisted FFR-CT and invasive FFR reached 0.81, demonstrating its feasibility as a non-invasive alternative in clinical practice (Xue et al., 2022).

Additionally, another investigation highlighted the advantages of AI-QCT in vascular stenosis assessment, emphasizing its utility in high-risk patients. AI-QCT, integrating multiple imaging modalities, not only provides anatomical information but also plays a crucial role in functional assessment, supporting clinical decision-making. These research findings indicate that AI platforms and software are playing increasingly important roles in cardiovascular disease diagnosis and treatment, with the potential to become integral components of routine clinical practice, providing more precise therapeutic strategies for patients.

Through these examples, we observe that AI-QCT technology applications not only enhance the accuracy of coronary lesion detection but also promote clinical workflow optimization and reduce physician workload. These achievements herald that widespread AI applications in medical imaging will bring revolutionary changes to cardiovascular disease diagnosis and management.

### 1.2 Clinical validation of AI-FFR technology diagnostic performance

In recent years, artificial intelligence-based coronary functional assessment technology, particularly AI-driven fractional flow reserve (AI-FFR), has garnered widespread attention for its potential in coronary artery disease (CAD) diagnosis. This technology provides non-invasive functional assessment by analyzing coronary angiographic images, facilitating improved clinical decision-making. Clinical validation of AI-FFR represents a critical step in ensuring its accuracy and reliability.

According to recent studies, the diagnostic performance of AI-FFR technology has been significantly validated. For instance, one study demonstrated that AI-FFR achieved sensitivity and specificity of 91% and 95%, respectively, in detecting coronary stenosis, with an overall accuracy of 94% (Luo et al., 2024). These results indicate that AI-FFR not only provides reliable diagnostic information in clinical practice but also completes calculations within a remarkably short timeframe (average 37.5 s), substantially enhancing clinical workflow efficiency (Chiou et al., 2024).

In comparison with invasive FFR, AI-FFR demonstrates superior performance, with results highly consistent with traditional pressure wire measurements. This consistency provides robust support for AI-FFR’s clinical application. For example, in a retrospective study comparing Murray law based QFR (μQFR) with invasive instantaneous wave-free ratio (iwFR), μQFR demonstrated moderate correlation (r = 0.47, p = 0.001) but showed promising diagnostic accuracy with an AUC of 0.84 for predicting functionally significant lesions. The sensitivity and specificity of μQFR (<0.8) were 89% and 74% respectively, suggesting its potential as a non-invasive tool for physiological assessment of coronary lesions (Samaan et al., 2024).

Furthermore, AI-FFR’s advantages extend beyond accuracy to include its capability in detecting different plaque types. Research indicates that AI-FFR can effectively identify high-risk plaques and associated hemodynamic changes, information that is difficult to obtain through conventional coronary angiography (Wu et al., 2025). This capability is particularly important as high-risk plaque identification enables physicians to implement preventive measures at early stages, thereby reducing cardiovascular event incidence.

Despite AI-FFR’s favorable performance in clinical validation, limitations and challenges in its application must be acknowledged. For instance, algorithm accuracy may be influenced by image quality and operational techniques, requiring clinical vigilance when utilizing AI-FFR. Additionally, widespread implementation and large-scale application of AI-FFR necessitate further empirical research and clinical trials to ensure its applicability and reliability across diverse patient populations.

#### 1.2.1 Comparative analysis with invasive FFR

Recently, the application of AI-based FFR technology in coronary computed tomography angiography (CCTA) has gained increasing attention. Studies demonstrate that AI-based CT-FFR can effectively simulate invasive FFR measurements for functional evaluation of coronary stenosis, with diagnostic accuracy of 0.82 and area under the curve of 0.82 on a per-patient level. This positions AI-based CT-FFR as a promising non-invasive tool for physiological assessment of coronary lesions, going beyond mere anatomical stenosis evaluation (Guo et al., 2024; Li B. et al., 2024).

In identifying functionally ischemic lesions, AI-FFR demonstrates high correlation with invasive FFR, with studies showing that AI-FFR can effectively reduce false-positive rates. Specifically, AI-FFR utilizes computational fluid dynamics and deep learning algorithms to perform in-depth CT image analysis, thereby more accurately assessing hemodynamic changes. This technological improvement significantly enhances clinical assessment capabilities for vascular stenosis, facilitating more personalized treatment planning (Wu et al., 2025; Li M. et al., 2024).

Moreover, AI-FFR application significantly reduces the need for invasive procedures, decreasing patient risk associated with invasive examinations. Studies indicate that among patients utilizing AI-FFR, only a minority require further invasive FFR examination, thereby reducing medical costs while improving patient safety and satisfaction (Roguin et al., 2021; Zhang et al., 2023). Therefore, AI-FFR demonstrates superior potential compared to traditional methods in clinical applications for assessing coronary stenosis, particularly in screening and determining functionally ischemic lesions.

Based on these research findings, we conclude that AI-FFR demonstrates excellent performance in accuracy, sensitivity, and specificity for identifying coronary lesions, showing high correlation with invasive FFR and providing new perspectives and directions for coronary heart disease diagnosis and treatment. With continued technological advancement and application expansion, AI-FFR is expected to play an increasingly important role in clinical coronary heart disease management.

#### 1.2.2 Correlation with quantitative coronary angiography (QCA)

The correlation between CT perfusion-based FFR (CTP-FFR) and quantitative coronary angiography (QCA) in assessing coronary stenosis severity has been evaluated across different stenosis categories. Studies have demonstrated that CTP-FFR shows varying diagnostic performance across mild (30%–49%), moderate (50%–69%), and severe (≥70%) stenosis groups as classified by QCA. The diagnostic accuracy of CTP-FFR showed promising results with an area under the curve (AUC) of 0.953, significantly outperforming traditional CCTA (AUC 0.830) in functional assessment (Gao et al., 2023). This suggests CTP-FFR’s potential value in evaluating coronary lesions, particularly in mild-to-moderate stenotic lesions, providing a non-invasive alternative for functional assessment of coronary stenosis.

Studies show that AI-FFR performs well in measurements. For example, in a study involving 463 patients with 600 vessels, using invasive FFR as the reference standard, AI-FFR achieved a diagnostic accuracy of 82% with an area under the curve of 0.82 on a per-patient level. For different clinical presentations, including chronic coronary syndrome and acute chest pain patients, AI-FFR demonstrated superior predictive capability for major adverse cardiac events compared to CCTA alone (Guo et al., 2024). Compared to QCA, AI-FFR better reflects myocardial ischemia when determining functional stenosis (Alsharqi and Edelman, 2025), especially in patients where traditional QCA assessment results are inconclusive.

Additionally, AI-FFR’s rapid computational capability represents one of its primary advantages. When processing large volumes of imaging data, AI-FFR completes calculations in merely 37 s, significantly improving workflow efficiency in clinical environments (Ben-Assa et al., 2023). This efficiency enables physicians to obtain accurate assessment results quickly, facilitating rapid treatment decisions.

In specific applications, the combination of AI-FFR and QCA enhances coronary lesion detection rates. Research indicates that AI-FFR performs excellently in detecting moderate and severe stenosis, particularly in complex coronary lesions where AI-FFR performance exceeds traditional QCA assessment (Griffin et al., 2023; Biondi-Zoccai et al., 2025; Khelimskii et al., 2024/09). Through this approach, AI-FFR provides important support for clinical coronary heart disease management.

#### 1.2.3 Comparison with myocardial perfusion imaging (MPI)

Artificial intelligence-assisted coronary computed tomography angiography (AI-QCT) demonstrates significant advantages over traditional myocardial perfusion imaging (MPI) in diagnosing coronary stenosis severity. According to the CREDENCE trial study of 301 patients (Lipkin et al., 2022), AI-QCT showed significantly higher diagnostic performance than MPI, with higher Area Under Curve (AUC) values for predicting ≥50% stenosis (0.88 vs. 0.66), ≥70% stenosis (0.92 vs. 0.81), and FFR <0.80 (0.90 vs. 0.71). AI-QCT also demonstrated superior sensitivity (95% vs. 74%) and specificity (63% vs. 43%) compared to MPI for detecting ≥50% stenosis. Furthermore, implementing AI-QCT in diagnostic algorithms could reduce invasive angiography utilization by up to 49%, potentially decreasing unnecessary downstream testing and associated costs.

In AI-QCT, artificial intelligence analysis enables quantitative and qualitative assessment of coronary plaques, better identifying high-risk plaques and thereby guiding clinical decisions. For example, AI-QCT can accurately assess plaque volume, composition, and blood flow impact—information crucial for determining the functional significance of coronary lesions. While MPI provides myocardial perfusion information, it often fails to analyze plaque characteristics in detail, resulting in incomplete myocardial ischemia assessment.

Furthermore, AI-QCT application significantly reduces misdiagnosis rates. Research reveals that CCTA has superior diagnostic performance than MPI (Budoff et al., 2017). AI-QCT, through precise computer algorithm analysis could elevate diagnostic accuracy and aids for treatment plans (Coma et al., 2024). Compared to traditional MPI, AI-QCT shows significant improvement in both diagnostic sensitivity and specificity. When detecting coronary stenosis of ≥50% or above, AI-QCT achieved 95% sensitivity and 63% specificity, compared to MPI’s 74% sensitivity and 43% specificity (Lipkin et al., 2022).

Overall, AI-QCT combined with FFR technology not only enhances coronary stenosis diagnostic efficiency but also reduces misdiagnosis rates. Compared to traditional myocardial perfusion imaging techniques, AI-QCT demonstrates superior clinical application value. This advancement provides new perspectives and methods for early diagnosis and treatment of coronary heart disease patients, holding significant clinical importance.

#### 1.2.4 Comprehensive review of multi-center large-scale study results

In coronary heart disease diagnosis and treatment, AI-FFR technology has been progressively validated as an effective tool, with its stability and broad applicability further confirmed through multi-center large-scale studies. Studies such as CREDENCE and PACIFIC-1 demonstrate AI-FFR’s reliability and accuracy in assessing coronary stenosis severity.

The CREDENCE study evaluated 600 vessels in 463 patients who underwent coronary CT angiography (CCTA) and invasive FFR measurements within 90 days. Results showed AI-FFR diagnostic accuracy of 0.82, with per-patient area under the curve (AUC) of 0.82, indicating good performance in functional assessment of coronary stenosis (Guo et al., 2024). Compared to traditional manual CT-FFR techniques, AI-FFR technology not only significantly reduced operation time but also achieved calculation success rates exceeding 99%. In chronic coronary syndrome and acute chest pain patients, AI-FFR demonstrated superior capability in predicting major adverse cardiac events compared to CCTA alone (Guo et al., 2024).

The PACIFIC-1 study further explored comparisons between coronary CT-FFR and other diagnostic tools, showing that AI-FFR performed excellently in identifying clinically significant stenosis, with sensitivity and specificity reaching 91% and 95%, respectively (Chiou et al., 2024). This indicates that AI-FFR can effectively distinguish lesions requiring clinical intervention, thereby assisting clinicians in making more precise treatment decisions.

Moreover, with continued technological advancement, AI-FFR’s application scope continues expanding. Research demonstrates that beyond excellent stability and accuracy, AI-FFR technology operates efficiently in actual clinical environments, providing new perspectives and methods for early coronary heart disease diagnosis and treatment (Jo et al., 2025; Fairbairn et al., 2025). These research achievements establish a foundation for widespread AI-FFR application, demonstrating its potential and importance in coronary heart disease assessment.

### 1.3 Clinical application value of AI-FFR technology in coronary heart disease management

AI-FFR technology has demonstrated significant application value in coronary artery disease (CAD) management. By extracting hemodynamic information from coronary computed tomography angiography (CCTA) data, AI-FFR can assess the functional significance of coronary stenosis non-invasively, thereby helping physicians make more precise clinical decisions. Compared with traditional invasive FFR measurements, AI-FFR not only enhances diagnostic efficiency but also reduces patient risk and discomfort.

Multiple studies indicate that AI-FFR demonstrates excellent accuracy in clinical practice. For instance, one study evaluating 600 vessels in 463 patients found that AI-FFR exhibited outstanding diagnostic performance, achieving 82% diagnostic accuracy at the per-patient level (area under the curve AUC of 0.82), significantly superior to traditional manual FFR techniques (Guo et al., 2024). These results suggest that AI-FFR technology not only enables rapid computation but also reduces procedural complexity, thereby improving clinical application convenience and practicality.

Another important application of AI-FFR technology is its prognostic assessment capability in CAD patients. Research indicates that AI-FFR technology more effectively predicts major adverse cardiovascular events (MACE) (Guo et al., 2024). In acute chest pain patients, AI-FFR’s predictive value substantially exceeds traditional methods, more accurately identifying patients requiring invasive intervention. Additionally, AI-FFR demonstrates high sensitivity and specificity across various coronary lesion types, particularly in identifying functional stenosis, with sensitivity reaching 91% and specificity of 95% (Ben-Assa et al., 2023).

In practical applications, AI-FFR technology’s advantages are further manifested in its ability to provide real-time lesion information, helping physicians make timely and reasonable decisions in complex clinical scenarios. With continued technological advancements, AI-FFR will integrate more cardiovascular imaging data, providing more comprehensive assessments. For example, AI models combining computed tomography (CT) and optical coherence tomography (OCT) data can achieve automated analysis of plaque characteristics and coronary function, providing more precise evidence for CAD management (Fairbairn et al., 2025).

#### 1.3.1 Optimizing diagnostic procedures and reducing invasive examinations

Artificial intelligence (AI)-assisted coronary CTA (computed tomography angiography) screening demonstrates significant advantages in CAD diagnosis, particularly in optimizing diagnostic procedures and reducing invasive examinations. Using AI technology effectively reduces unnecessary invasive coronary angiography, thereby lowering medical costs and patient risks. AI-FFR (fractional flow reserve) technology, by combining automated coronary plaque segmentation and lumen extraction models, significantly improves diagnostic efficiency. For example, studies show that AI-based fully automated CT-FFR technology reduces operation time threefold compared to traditional manual methods, providing a more convenient and objective tool for functional assessment of coronary stenosis in clinical environments (Guo et al., 2024).

According to one study, AI-FFR achieves 82% diagnostic accuracy in patients and, compared with traditional methods, reduces the number of invasive examinations patients need to undergo without increasing additional risks (Li B. et al., 2024). Further research indicates that AI-FFR technology not only demonstrates high accuracy but also exhibits superior predictive capability in cardiovascular risk assessment compared to traditional methods relying solely on CCTA, providing clinicians with more scientific decision-making evidence (Wu et al., 2025).

When managing acute chest pain or chronic coronary syndrome patients, AI-FFR technology rapidly provides clinicians with critical functional assessments, avoiding unnecessary invasive examinations and reducing patient discomfort and psychological burden during the diagnostic process, which holds significant importance for optimizing overall patient treatment experience (Li M. et al., 2024). Additionally, AI-FFR application effectively improves patient quality of life and reduces subsequent medical expenses caused by insufficient detection or misdiagnosis, further alleviating economic burden on healthcare systems (Ayoub et al., 2024).

#### 1.3.2 Improving accuracy of early diagnosis and risk stratification in coronary heart disease

Artificial intelligence-based quantitative analysis combined with hemodynamic assessment supports more precise functional lesion determination, which is crucial for improving early diagnosis accuracy and risk stratification in coronary heart disease. In recent years, CT-FFR, as a non-invasive assessment technique, has been increasingly applied in clinical practice, particularly in coronary artery disease (CAD) diagnosis. Research indicates that CT-FFR effectively evaluates the functional significance of coronary stenosis, thereby helping physicians formulate individualized treatment plans (Guo et al., 2024).

Utilizing artificial intelligence technology, the CT-FFR calculation process becomes more efficient and accurate. For example, a recent study proposed a fully automated AI-based CT-FFR technology that significantly improved calculation speed and accuracy through automated coronary plaque segmentation and lumen extraction models combined with simplified three-dimensional computational fluid dynamics (Guo et al., 2024). This technology demonstrated 0.82 diagnostic accuracy in a study of 463 patients, with calculation success rates exceeding 99%, indicating its enormous potential in clinical practice.

Further research also indicates that CT-FFR better predicts major adverse cardiac events compared to traditional invasive methods, especially in chronic coronary syndrome and acute chest pain patients (Guo et al., 2024). By combining CT-FFR with plaque characteristics, physicians can better identify high-risk plaques and develop more effective interventions, thereby optimizing patient prognosis (Wu et al., 2025).

Additionally, other emerging technologies, such as quantitative flow ratio (QFR) and optical flow ratio (OFR), provide new approaches for coronary hemodynamic assessment. These technologies utilize medical imaging techniques like coronary angiography and computed tomography, combined with artificial intelligence algorithms, to rapidly and non-invasively evaluate the functional significance of coronary stenosis (Li B. et al., 2024). The advantages of this approach lie in its efficiency and accuracy, enabling rapid identification of high-risk patients in clinical practice, thereby improving early diagnosis accuracy and timeliness.

#### 1.3.3 Guiding individualized treatment decisions

In clinical treatment of coronary heart disease, accurate identification of functional stenosis is key to formulating individualized treatment decisions. Traditionally, functional stenosis assessment relied on invasive methods such as measuring coronary FFR. However, with technological advancements, artificial intelligence-based coronary CT angiography (CCTA)-derived fractional flow reserve (CT-FFR) technology has emerged. This technology not only non-invasively assesses coronary functional stenosis but also significantly improves diagnostic efficiency while reducing patient risk.

Research indicates that CT-FFR demonstrates excellent diagnostic performance in functional stenosis assessment. For example, in a large-scale prospective study, CT-FFR achieved 82% diagnostic accuracy, providing strong support for clinical decision-making (Guo et al., 2024). Compared with traditional FFR measurements, CT-FFR not only simplifies operation but also substantially reduces operation time, decreasing patient burden. The successful application of this technology provides new ideas and evidence for individualized treatment decisions in coronary heart disease patients, enabling physicians to more precisely select interventional or pharmacological treatment plans.

Furthermore, artificial intelligence technology application enables more comprehensive assessment of patient coronary stenosis characteristics, including plaque composition and morphology. This information is crucial for determining plaque stability and rupture risk, thus helping physicians formulate more personalized treatment plans. For instance, combining CT-FFR with plaque characteristic analysis identifies high-risk plaque features such as thin fibrous caps and large lipid cores, which are closely associated with cardiovascular events (Wu et al., 2025). Therefore, by integrating CT-FFR with plaque characteristic analysis, clinicians can more effectively identify patients requiring interventional treatment and formulate individualized treatment plans.

Additionally, CT-FFR application provides patients with more convenient treatment options, particularly in early assessment of acute coronary syndromes. Through non-invasive methods, CT-FFR rapidly evaluates patient hemodynamic status, helping physicians quickly decide whether further interventional or pharmacological treatment is needed (Li B. et al., 2024). This is particularly important in emergency care for acute chest pain patients, where timely decisions significantly improve survival rates.

#### 1.3.4 Predicting patient prognosis and adverse event risk

AI-FFR models, as an emerging technology, have demonstrated significant clinical value in predicting major adverse cardiovascular events (MACE). By integrating clinical risk factors, AI-FFR models provide more precise prognostic assessments, supporting long-term management of coronary heart disease patients. Research indicates that AI-FFR shows significant improvements in both accuracy and efficiency compared to traditional fractional flow reserve (FFR) technology. For example, comparison between AI-FFR and invasive FFR measurement results shows that AI-FFR achieves 91% sensitivity and 95% specificity, effectively identifying functional myocardial ischemia (Ben-Assa et al., 2023).

In a study examining AI-FFR diagnostic performance, data from 463 patients who underwent coronary CT angiography (CCTA) and FFR detection were analyzed. Results showed that AI-FFR’s predictive value for identifying major adverse cardiovascular events surpassed assessments solely relying on CCTA, particularly in chronic coronary syndrome and acute chest pain patients. Additionally, the area under the curve (AUC) reached 0.82, indicating the model’s effectiveness in clinical applications (Guo et al., 2024).

AI-FFR models integrating clinical risk factors provide more comprehensive assessment of patient prognosis and adverse event risk. This integration not only enhances predictive capability for cardiovascular events but also provides reliable evidence for formulating individualized treatment plans. For instance, research indicates that in chronic coronary heart disease patients, AI-FFR serves as an objective tool providing clinicians with in-depth insights into coronary functional status, thereby optimizing treatment strategies (Xue et al., 2022).

Further research also reveals associations between AI-FFR and various clinical characteristics, demonstrating its potential in identifying high-risk patients. Researchers emphasize that AI-FFR model application provides more precise cardiovascular event risk assessment without increasing patient burden, helping clinicians formulate more reasonable interventions, thereby improving patients’ long-term prognosis (Carvalho et al., 2024).

### 1.4 Challenges and limitations of AI-FFR technology

As early diagnosis and treatment of coronary artery disease gains increasing attention, artificial intelligence (AI)-driven fractional flow reserve (FFR) technology derived from coronary CT angiography (CCTA) has garnered significant interest. While this technology’s rapid development provides new possibilities for non-invasive hemodynamic assessment, it still faces numerous challenges and limitations in practical application.

First, the accuracy and reliability of AI-FFR technology require further validation. Although certain studies suggest AI-FFR demonstrates good sensitivity and specificity in diagnosing functional stenosis, AI system performance may be limited in complex lesions. For example, AI-FFR assessment of severely calcified lesions may be less accurate than traditional pressure wire measurements (Fairbairn et al., 2025). Furthermore, while AI-FFR effectively predicts flow reserve in most cases, its accuracy may decrease in specific patient populations, such as diabetic or elderly patients, necessitating clinical caution when interpreting results.

Second, the universality and acceptance of AI technology in clinical applications require improvement. Despite AI-FFR technology’s potential to reduce invasive procedures and lower medical costs, its application in actual clinical environments is constrained by complexity and technical requirements (Jo et al., 2025). Many hospitals and physicians may lack sufficient resources or training to effectively implement this new technology, necessitating appropriate training and education systems to enhance clinicians’ understanding and application capabilities of AI-FFR technology.

Third, AI-FFR implementation depends on high-quality imaging data, and data acquisition and processing may affect final results. CCTA image quality, lesion anatomical location, and patient physiological status can all influence AI algorithm performance (Chiou et al., 2024). Therefore, ensuring image quality and standardized imaging protocols is particularly important, requiring continuous technical investment and improvement from medical institutions.

Finally, despite AI-FFR technology’s promising clinical prospects, ethical, legal, and privacy issues urgently need resolution. For instance, addressing patient data privacy concerns and determining circumstances for legal responsibility are challenges that must be confronted when promoting AI technology (De Filippo et al., 2025). Consequently, relevant policies and regulations must evolve with technological developments.

#### 1.4.1 Image quality dependence and data heterogeneity

The quality of CTA images is crucial for accurate analysis by artificial intelligence (AI)-based models. Image quality directly affects AI model effectiveness in identifying coronary stenosis severity. According to existing research, coronary CTA image quality is influenced by multiple factors, including scanning equipment performance, image acquisition techniques, and patient physiological characteristics. For example, scanning equipment resolution, exposure time, and image post-processing techniques all affect final image clarity and detail presentation (Carvalho et al., 2024).

Among different patients, variations in anatomical structures, pathological complexity, and presence of comorbidities similarly increase data heterogeneity. Research indicates that patient body habitus, age, and cardiovascular health status all lead to differences in coronary CTA image presentation (Guo et al., 2024). Therefore, AI models must consider these diverse imaging data during training to provide reliable diagnostic results in clinical applications.

Furthermore, artificial intelligence technology development has made coronary CTA image analysis more efficient. Research has proposed an AI-based fully automated CT-FFR technology that combines automated coronary plaque segmentation and lumen extraction models, generating high-quality functional assessment results in a short time and demonstrating high accuracy across different patients (Ben-Assa et al., 2023). However, this technology’s effectiveness still depends on input image quality; therefore, ensuring high-quality image acquisition and processing is a prerequisite for AI-assisted diagnosis.

Future research should focus on combining multiple imaging techniques to improve image quality and consistency, thereby enhancing AI model training effects and clinical application accuracy. By establishing standardized image quality assessment systems, clinical practice can be better guided, ensuring accurate identification of coronary stenosis severity and effective intervention (Ben-Assa et al., 2023).

#### 1.4.2 Algorithm generalizability and external validation requirements

In research using artificial intelligence (AI) technology for coronary CTA (computed tomography) vascular stenosis assessment, algorithm generalizability and external validation are crucial. Studies show that algorithm performance may vary significantly across different populations and clinical environments. These variations affect not only algorithm applicability in specific populations but also its practical application in broader populations (Guo et al., 2024; Carvalho et al., 2024).

To improve algorithm generalizability, multi-center and multi-population validation is essential. This validation process can be achieved by collecting patient data from different regions, ethnic backgrounds, and clinical conditions. For example, in one multi-center study, automated CT-FFR (computed tomography-derived fractional flow reserve) technology demonstrated good diagnostic accuracy across multiple hospitals in China and was validated across different patient populations (Xue et al., 2022; Ben-Assa et al., 2023). This research demonstrated that algorithm consistency and reliability across different centers and patient populations provide a foundation for wider application of this technology.

Additionally, external validation can assess algorithm effectiveness in actual clinical environments. For instance, in a study of acute coronary syndrome patients, comparing data from different hospitals validated AI algorithm effectiveness in predicting cardiovascular events, providing important evidence for clinical application (Liu et al., 2023; Peivandi et al., 2023). However, the challenge of external validation lies in potential algorithm performance variations due to clinical environment differences, requiring researchers to consider these potential influencing factors when designing validation studies.

#### 1.4.3 Computational resource and real-time limitations

In artificial intelligence applications combining coronary CTA (computed tomography angiography) with FFR, computational resource requirements and real-time limitations present two significant challenges. In recent years, with the rapid development of medical imaging technology, particularly advances in three-dimensional reconstruction and blood flow simulation, coronary CTA applications have become increasingly widespread. However, high-precision three-dimensional reconstruction and blood flow dynamic simulation require substantial computational resources, creating certain limitations in actual clinical applications.

For example, although current AI-driven CT-FFR technology demonstrates good diagnostic performance clinically, its computation process remains complex and time-consuming. One study indicates that traditional CT-FFR methods often require lengthy processing times, while newly developed fully automated CT-FFR technology can reduce operation time to one-third of the original, with calculation success rates exceeding 99% (Guo et al., 2024). Nevertheless, real-time application of this technology still faces challenges, particularly in high-workload clinical environments where physicians need rapid results to support decision-making.

Furthermore, non-invasive FFR estimation relies on complex computational models that process large volumes of imaging data and perform real-time hemodynamic analysis. This analysis requires not only powerful computational capabilities but also high-precision image segmentation and feature extraction, which are often limited by equipment performance and algorithm efficiency in practical operations. Research shows that functional assessments using artificial intelligence technology significantly improve diagnostic accuracy, but computational resource bottlenecks still limit widespread application (Carvalho et al., 2024; Farhad et al., 2023).

In clinical practice, balancing real-time requirements and computational resource needs becomes an urgent problem requiring resolution. To achieve widespread clinical application, future research may need to focus on improving algorithm efficiency and computational speed while optimizing hardware facilities to support more complex computational tasks. Through these improvements, we hope to enhance response speed to acute coronary events while maintaining diagnostic accuracy, providing patients with more timely treatment plans. These efforts will help advance practical applications of artificial intelligence technology in coronary heart disease diagnosis and treatment, thereby improving patient prognosis and quality of life.

#### 1.4.4 Technical implementation challenges

##### 1.4.4.1 Computational infrastructure requirements for AI-FFR systems

The implementation of AI-FFR technology demands robust computational infrastructure that many healthcare facilities find challenging to establish and maintain. High-performance computing systems are essential for processing complex coronary CT images and running sophisticated AI algorithms in real-time. These systems typically require specialized Graphics Processing Units (GPUs) and powerful processors capable of handling intensive computational tasks (Ben-Assa et al., 2023).

Storage requirements present another significant challenge, as high-resolution medical imaging data can consume substantial space. A typical cardiac CT scan can generate several gigabytes of data, and when multiplied by thousands of patients, the storage needs become enormous. Healthcare facilities must invest in scalable storage solutions while ensuring quick data retrieval capabilities (Paul et al., 2022).

For smaller healthcare facilities. The initial investment in hardware, ongoing maintenance costs, and regular upgrades represent significant financial commitments. Cloud-based solutions offer an alternative, but they come with their own challenges regarding data security, bandwidth requirements, and monthly subscription costs. Additionally, facilities must consider redundancy and backup systems to prevent service interruptions, further adding to the infrastructure complexity (Peters et al., 2024).

##### 1.4.4.2 Integration challenges in AI-FFR implementation

The integration of AI-FFR systems into existing hospital infrastructures presents significant technical and operational challenges. Many healthcare facilities operate with legacy systems that may use different data formats and communication protocols, making seamless integration complicated. For instance, DICOM (Digital Imaging and Communications in Medicine) standards, while widespread, may not fully support all AI-FFR specific requirements, necessitating additional interface development (Ben-Assa et al., 2023).

Workflow integration poses another crucial challenge. Healthcare providers often need to modify their established clinical workflows to accommodate AI-FFR analysis steps, which can create efficiency bottlenecks (Costantini et al., 2024). Additionally, ensuring real-time data synchronization between the AI-FFR system and hospital electronic health records (EHR) systems requires robust middleware solutions.

The requirement for backup systems and redundancy adds another layer of complexity. Healthcare facilities must maintain uninterrupted service availability, necessitating redundant systems and regular backup protocols (Zreik et al., 2019). This includes maintaining backup servers, implementing failover systems, and ensuring data recovery capabilities, all while maintaining compliance with healthcare data security regulations.

##### 1.4.4.3 Clinical validation barriers in AI-FFR implementation

The validation of AI-FFR technology faces several significant hurdles in the clinical setting. Large-scale validation studies, crucial for establishing the technology’s reliability, are often hampered by limited patient populations and inconsistent data collection methods across different healthcare facilities (Nakazato et al., 2013).

Multi-center trials, while essential for comprehensive validation, present unique challenges. These include variations in imaging protocols, equipment specifications, and clinical practices across different institutions. Standardizing these elements while maintaining data quality and consistency requires extensive coordination and resources (Futoma et al., 2020).

The cost implications of conducting thorough validation studies are substantial. Beyond financial investments, these studies demand significant time commitments from healthcare professionals and researchers. Many facilities struggle to allocate resources for such extensive research while maintaining regular patient care services (Futoma et al., 2020).

Long-term outcome studies, necessary for evaluating the technology’s impact on patient health over time, are particularly challenging. These studies require sustained funding, consistent patient follow-up, and careful documentation of outcomes over several years, making them difficult to execute effectively (Nørgaard et al., 2014).

#### 1.4.5 Regulatory oversight and clinical acceptance

With the rapid development of artificial intelligence (AI) technology in healthcare, regulatory oversight and clinical acceptance of AI medical products have gradually gained attention. First, AI medical products must comply with strict regulatory standards to ensure their safety and effectiveness. Currently, many countries and regions, particularly the European Union and United States, have begun establishing relevant laws and regulations requiring thorough clinical validation and safety assessment of AI products before market entry. For example, the European Union’s Medical Device Regulation (MDR) imposes higher requirements on AI-driven medical devices to prevent medical accidents caused by technical defects.

However, despite these regulations, clinical acceptance of AI medical products still faces challenges. On one hand, clinical physicians often demonstrate low trust and acceptance of AI-assisted diagnosis, primarily due to insufficient understanding of AI technology and skepticism regarding its clinical effectiveness. Many physicians may develop dependency on AI tools when using them, especially in complex clinical judgment situations (Kobayashi, 2024). For instance, when performing imaging diagnostics, physicians may blindly trust AI-provided results while neglecting their professional judgment. This phenomenon not only affects clinical decision-making quality but may also increase patient safety risks.

Additionally, transparency and explainability of AI products are important factors affecting clinical acceptance. Many physicians express that when using AI systems, they hope to understand the AI decision-making process to better combine AI recommendations with their clinical experience (Beckers et al., 2021; Winder et al., 2024). However, many current AI systems still have “black box” issues, making it difficult for physicians to understand decision-making bases, which to some extent reduces their trust. To improve clinical acceptance of AI medical products, relevant departments and developers need to implement measures enhancing transparency and explainability. For example, providing detailed product descriptions, clinical validation data, and use cases can help healthcare professionals rates and acceptance in clinical settings (Jain et al., 2024; Obuchowicz et al., 2025).

### 1.5 Future directions and research trends

AI- FFR technology is emerging as a critical area of research and application in coronary heart disease diagnosis and treatment. With the rapid advancement of artificial intelligence and machine learning technologies, numerous recent studies have demonstrated their application potential in coronary computed tomography angiography (CCTA). For instance, latest research indicates that AI-driven CT-FFR technology exhibits excellent performance in providing objective and convenient tools for functional assessment of coronary stenosis, particularly in patients with chronic coronary syndrome and acute chest pain (Guo et al., 2024; Carvalho et al., 2024). The introduction of this novel technology not only enhances diagnostic accuracy but also substantially reduces operational time, making this technology more feasible and efficient in clinical applications.

Future developmental trends are primarily manifested in several aspects. First, artificial intelligence technology will continue to play a pivotal role in CT-FFR computational models. Researchers are exploring how to integrate deep learning algorithms with CT images to improve computational precision and efficiency. Studies demonstrate that using artificial intelligence for coronary CTA image analysis can provide consistent diagnostic performance across various types of coronary lesions (Xue et al., 2022). In the future, with continuous algorithm optimization, the application scope of CT-FFR technology is expected to further expand, not only limited to diagnosis but potentially playing a more significant role in treatment decision-making.

Second, with advancements in medical imaging technology, models combining machine learning and deep learning will become new tools for coronary heart disease management. These models can process more complex datasets, thereby enhancing clinical decision support capabilities (Tesche and Gray, 2020; Ploscaru et al., 2022). Simultaneously, with improvements in data acquisition techniques, healthcare professionals will be better able to integrate clinical data with imaging data to provide personalized treatment plans for patients.

Furthermore, as understanding of coronary heart disease and its related complications deepens, non-invasive assessment techniques based on FFR will gain more widespread application. Current research indicates that FFR-CT technology can not only improve diagnostic capabilities for coronary lesions but also provide important information in assessing myocardial ischemia risk, offering new evidence for developing clinical treatment protocols (Takami et al., 2025). In the future, with deeper research into coronary heart disease biomarkers, FFR technology is expected to combine with other biomarkers to form a more comprehensive assessment system.

Finally, with global emphasis on cardiovascular diseases, relevant policies and guidelines are continually being updated to support clinical practices based on the latest technologies. Researchers need to attend to feedback from clinical practice and incorporate this feedback into future research, thereby promoting the popularization and application of AI-based FFR technology in coronary heart disease diagnosis and treatment (Carvalho et al., 2024; Ben-Assa et al., 2023). Through these efforts, more efficient and precise coronary heart disease management can be achieved in the future, ultimately improving patients’ clinical outcomes.

## 2 Conclusion

With continuous advancements in medical technology, AI-FFR combined with coronary CT angiography (CTA) has introduced new opportunities for the diagnosis and management of coronary artery disease. The emergence of this technology has not only elevated the non-invasive diagnostic capabilities for coronary heart disease but also provided clinicians with more precise and efficient tools for identifying coronary stenosis. Through the aggregation of multicenter clinical research data, we observe AI-FFR’s excellent performance in sensitivity and specificity, demonstrating high concordance with traditional invasive FFR and quantitative coronary angiography results, indicating its broad potential in clinical applications.

The advantages of AI-FFR technology lie in its ability to optimize coronary heart disease diagnosis and treatment protocols, reducing unnecessary invasive procedures, thereby lowering patient risk and healthcare costs. Additionally, this technology provides robust support for individualized treatment and prognostic assessment, enabling clinicians to develop more precise treatment plans based on specific patient conditions. This transformation has not only improved patient satisfaction but also significantly promoted the standardization of coronary heart disease management.

However, despite AI-FFR technology’s numerous advantages, it still faces several challenges in practical applications. These include dependence on image quality, algorithm generalizability issues, and computational resource requirements, all of which may affect its implementation and dissemination. Addressing these challenges, future research should focus on multimodal data integration, model optimization, and big data technology applications to enhance AI-FFR’s reliability and universal applicability.

Overall, AI-based FFR technology represents a significant advancement in precision diagnosis and treatment of coronary heart disease. Promoting its standardized application can not only improve coronary disease management but also provide safer and more efficient medical services for patients. As technology continues to advance and clinical validation deepens, AI-FFR shows promise in becoming the “gold standard” for coronary heart disease diagnosis, opening new prospects for early identification and intervention in cardiovascular disease. We anticipate witnessing the widespread application of this technology across different healthcare settings and its positive impact on improving global cardiovascular health levels.

These developments mark a significant step forward in cardiovascular medicine, though continued research and validation will be crucial for optimizing its clinical implementation. The integration of AI-FFR into routine clinical practice represents a paradigm shift in coronary artery disease management, potentially revolutionizing how we approach cardiovascular care in the future.
